# Maintenance of Muscle Mass and Cardiorespiratory Fitness to Cancer Patients During COVID-19 Era and After SARS-CoV-2 Vaccine

**DOI:** 10.3389/fphys.2021.655955

**Published:** 2021-06-25

**Authors:** Miguel S. Conceição, Sophie Derchain, Felipe Cassaro Vechin, Guilherme Telles, Guilherme Fiori Maginador, Luís Otávio Sarian, Cleiton Augusto Libardi, Carlos Ugrinowitsch

**Affiliations:** ^1^School of Physical Education and Sport, University of São Paulo, São Paulo, Brazil; ^2^Department of Obstetrics and Gynecology, Faculty of Medical Sciences, University of Campinas, Campinas, Brazil; ^3^MUSCULAB-Laboratory of Neuromuscular Adaptations to Resistance Training, Department of Physical Education, Federal University of São Carlos, São Carlos, Brazil

**Keywords:** COVID-19, cancer, exercise, physical training, physical distance

## Abstract

There is emerging evidence that decreased muscle mass and cardiorespiratory fitness (CRF) are associated with increased risk of cancer-related mortality. This paper aimed to present recommendations to prescribe effective and safe exercise protocols to minimize losses, maintain or even improve muscle mass, strength, and CRF of the cancer patients who are undergoing or beyond treatment during the COVID-19 era. Overall, we recommend performing exercises with bodyweight, elastic bands, or suspension bands to voluntary interruption (i.e., interrupt the exercise set voluntarily, according to their perception of fatigue, before concentric muscular failure) to maintain or increase muscle strength and mass and CRF during COVID-19 physical distancing. Additionally, rest intervals between sets and exercises (i.e., long or short) should favor maintaining exercise intensities between 50 and 80% of maxHR and/or RPE of 12. In an exercise program with these characteristics, the progression of the stimulus must be carried out by increasing exercise complexity, number of sets, and weekly frequency. With feasible exercises attainable anywhere, modulating only the work-to-rest ratio and using voluntary interruption, it is possible to prescribe exercise for a wide range of patients with cancer as well as training goals. Exercise must be encouraged; however, exercise professionals must be aware of the patient’s health condition even at a physical distance to provide a safe and efficient exercise program. Exercise professionals should adjust the exercise prescription throughout home confinement whenever necessary, keeping in mind that minimal exercise stimuli are beneficial to patients in poor physical condition.

## Introduction

The novel 2019 coronavirus disease (COVID-19) pandemic is a global public health emergency. Despite vaccines having been approved, it will take a long time to immunize a sufficient percentage of the population. Non-pharmaceutical interventions represent the most effective strategy to control the COVID-19 and the SARS-CoV-2 spread until global immunization. Strategies such as wearing a mask, having good personal hygiene, and maintaining physical distance have been deemed effective measures to limit spread (Kantor and Kantor, 2020), especially before the SARS-CoV-2 vaccine. Even though social life is gradually returning to normal after the SARS-CoV-2 vaccine, home confinement and physical distance will continue to be encouraged, mainly for people at increased risk for COVID-19, including patients with cancer and cancer survivors. While avoiding contagion, these measures have caused negative health-related behavioral changes. For instance, people in home confinement decrease the accumulated minutes of physical activity per week (Stanton et al., 2020), leading to drops in muscle strength (Papiol et al., 2016) and mass (Kyle et al., 2004) (i.e., myocyte atrophy). It has also been shown that reduced levels of physical activity lead to a ∼7% decrease in cardiorespiratory fitness (CRF) (i.e., VO_2__max_) in young males (Krogh-Madsen et al., 2010). The decline in muscle mass and VO_2__max_ are clinically relevant, as they are independently associated with reduced quality of life and increased mortality, especially in individuals undergoing disease-related treatments, including cancer treatment based on chemotherapy.

Cancer treatment based on chemotherapy has decreased the number of disease-related deaths and increased longevity (Early Breast Cancer Trialists’ Collaborative Group, 2005; Early Breast Cancer Trialists’ Collaborative Group, Darby et al., 2011); nonetheless, it can greatly reduce muscle strength, muscle mass, and CRF, which increase the risk of death (Psutka et al., 2014; Reisinger et al., 2015; Guinan et al., 2018; Wang et al., 2018) and cancer recurrence and reduce quality of life (Nipp et al., 2017; Chang et al., 2018). The prevalence of low muscle mass is 16% among long-term breast cancer survivors (Villasenor et al., 2012) and 56 and 60% among women and men, respectively, with colorectal cancer (Broughman et al., 2015). Additionally, 50% of metastatic breast cancer patients with sarcopenia present capecitabine toxicity, compared to 20% of those without sarcopenia (Griffin et al., 2019). Regarding the decrease in CRF, there is emerging evidence that cardiomyocyte atrophy and fibrosis are the main factors leading to decreased cardiac function and, consequently, CRF. Cancer-related factors associated with decreased cardiac function are reduced physical activity levels, myocyte atrophy, tumor-released pro-cachectic factors, inflammatory and neuronal modulators, and chemotherapy-induced cardiotoxicity (Ausoni et al., 2020).

The conservation of skeletal and cardiac muscle mass and function predicts a better treatment response (Kazemi-Bajestani et al., 2016; Scott et al., 2018) as it prevents creating a vicious cycle in which loss of muscle mass and strength induces cardiac dysfunction, and this causes further decline in muscle mass and CRF (Clauss et al., 2017; Ausoni et al., 2020). Considering its importance, it is crucial to develop strategies to preserve skeletal and cardiac muscle mass and function for patients undergoing or beyond cancer treatment during COVID-19 pandemic physical distancing and eventually home confinement even after the SARS-CoV-2 vaccine.

Exercise training is a safe and efficient method to preserve and/or augment muscle mass and strength and CRF (Acsm, 2011; Hayes et al., 2019). Exercises on machines or using free-weights at moderate- to high-intensity (∼50–85% of the load that can be lifted just once with proper form, or one repetition maximum [1-RM]) have been recommended to increase muscle mass. Regarding CRF, exercises such as jogging, running, cycling, and swimming performed in continuous or interval modes are recommended, with moderate to high intensity (>65% of the VO_2_max), to significantly increase CRF (Acsm, 2011; Hayes et al., 2019). Traditionally, the implementation of exercise protocols depends on specialized equipment usually found in gyms and health centers. However, individuals restricted by home confinement or strict physical distance protocols due to COVID-19 cannot access those places. Alternatively, home-based exercises can adequately replace traditional exercise protocols by adjusting the implements, load, and structure of the exercises.

Prescribing exercise protocols during the COVID-19 and after the SARS-CoV-2 vaccine era of physical distancing and home confinement is challenging (Khoramipour et al., 2021). The exercise prescription has to be determined based on the identification of the goals, general and cancer-specific health issues, and their contribution to the risk of morbidity and/or mortality of the patient. Specific adjustments of the training protocol to the type of cancer and conditions associated with the treatment are beyond the scope of the present work, as they have been extensively discussed elsewhere (van der Leeden et al., 2018; Hayes et al., 2019). Thus, the objective of the present work is to present recommendations to prescribe effective and safe exercise protocols to minimize losses, maintain or even improve muscle mass, strength, and CRF depending on the condition of the cancer patients who are undergoing or beyond treatment during the COVID-19 era.

## Exercise Recommendations for Cancer Patients During the COVID-19 Era and After SARS-CoV-2 Vaccine: A Light at the End of the Tunnel

Exercise recommendations to maintain or increase muscle mass in cancer patients are similar to those suggested for healthy individuals. It is recommended to perform resistance exercises on machines or using free weights. Approximately 1–4 sets of 8–12 repetitions (i.e., moderate to high intensity, ∼50–85% of 1-RM) for 8–10 exercises targeting the major muscle groups, with rest intervals of 1–3 min between sets and exercises, should be performed at least once per week (Acsm, 2011; Campbell et al., 2019). It is also recommended that individuals progress from a minimal stimulus (e.g., one set and twice a week) to greater stimuli according to their adaptation and tolerance to exercise. This information is crucial for cancer patients who are cleared to exercise, but with short-term disease-associated conditions which are not favorable to exercise. While performing a low weekly volume promotes gains in strength and muscle mass (Singh et al., 2018), recent meta-analyses showed a dose-response relationship in which higher ranges of weekly sets (1–5, 5–9, and ≥10 sets) enhanced the gains in muscle strength (Ralston et al., 2017) and mass (Schoenfeld et al., 2016) for healthy individuals. These findings show that performing more than the recommended weekly sets (Schoenfeld et al., 2016; Ralston et al., 2017) may be beneficial for cancer patients who exercise regularly and are in good physical condition. Although these recommendations are feasible at home, they require machines or free weights to exercise with moderate to high intensities. It has recently been reported that low intensity (e.g., 30–50% 1-RM) exercise sets performed to concentric muscle failure (i.e., inability to complete another repetition with appropriate form) or close to failure produces similar gains in muscle strength and mass as moderate and high intensities (e.g., 50–80% of the 1-RM) (Nobrega et al., 2018; Santanielo et al., 2020). These adaptations occur irrespective of the manipulation of additional training variables (e.g., repetition duration, type of contraction, or exercise) (Nobrega et al., 2018; Damas et al., 2019; Soligon et al., 2020). Determining whether individuals are exercising close to concentric muscle failure is straightforward. They should be instructed to interrupt the exercise set voluntarily, according to their perception of fatigue, before concentric muscular failure (Santanielo et al., 2020). In fact, it has been recently shown that from these instructions, individuals interrupt exercise sets between 1 and 3 repetitions before concentric muscle failure (Nobrega et al., 2018; Santanielo et al., 2020). Voluntary interruption is practical and can also be used when performing body weight, elastic resistance band, and suspension band exercises at home. Importantly, these exercises can preserve and even increase muscle mass and strength, making them a viable option for cancer patients with no access to dumbbells, barbells, or exercise machines typically found in gyms.

Bodyweight exercises use different body positions as a means of resistance to perform work against gravity. These exercises can be easily prescribed and guided remotely using spreadsheets with photos, video clips, video calls, e-mails, or smartphone applications (van der Kolk et al., 2019). Studies comparing the effects of exercises using bodyweight (e.g., push-ups) with equipment exercises (e.g., bench press) showed similar increases in muscle mass and strength when both were performed to concentric muscle failure (Calatayud et al., 2015; Kikuchi and Nakazato, 2017). Elastic resistance bands have several levels of resistance (e.g., from 4.5 to 79 kg – super bands) (Lopes et al., 2019), allowing precise adjustment of the exercise load in a wide variety of exercises. The most common types of bands are tube bands with handles, super bands, loop bands (aka giant rubber bands), and therapy bands (de Oliveira et al., 2017). Several studies have shown that elastic resistance bands are effective in improving muscle mass and strength in young, middle-aged, and older adults with gains comparable to machines and free weights (de Oliveira et al., 2017; Liao et al., 2018, 2020; Lopes et al., 2019). Like elastic resistance bands, suspension bands can be performed in small rooms with adequate control of the exercise load. This exercise mode was specifically designed to train body segments (e.g., upper or lower limbs) while suspending the body in hanging straps, creating an unstable environment, using bodyweight and gravity to perform multiplanar and multijoint exercises (Mok et al., 2015). Recently, we did not find differences in muscle mass and strength gains between traditional resistance training machines and suspension band exercises in older individuals (Soligon et al., 2020).

As previously described, to maintain and increase muscle strength and mass, the recommendations for improving CRF of cancer patients are similar to those for healthy individuals. Exercise guidelines recommend jogging, running, cycling, and swimming, continuously or intermittent, with intensities based on a percentage of either VO_2__max_, maximal heart rate (%maxHR), or rate of perceived exertion (RPE). Exercise intensity should be between 60 and 80% VO_2__max_, 40 and 85% maxHR, or 12–14 RPE (6–20 scale), for 15–60 min per training session, and with a frequency of 2–7 sessions per week (Haskell et al., 2007; Acsm, 2011). Although low-intensity exercise performed for only 20 min and twice a week increases CRF, greater gains can be obtained by manipulating the training-related variables (Singh et al., 2018; Hayes et al., 2019; Maginador et al., 2020). For instance, recent studies have shown that exercise can be safely performed at even greater intensities during chemotherapy. For example, women with breast cancer performed interval training (7 efforts of 1-min at 90% VO_2__max_ interspaced by 2-min low-intensity intervals) for 8 weeks (Lee et al., 2019) and presented a significant increase in CRF. Adherence to the training protocol was excellent (i.e., 100% retention), and no adverse effects were reported. A recent meta-analysis (Wallen et al., 2020) showed that despite the short session duration, interval training protocols with short bouts (≤5 min) of high-intensity (80–100% maxHR), interspersed with passive or lower intensity active recovery periods, promote CRF gains comparable to continuous protocols (e.g., ≥20 min) of moderate-intensity (50–70% of maxHR) for patients with cancer or cancer survivors. However, we recently showed that this appears to occur in patients undergoing chemotherapy only when either continuous or interval exercise is performed at high intensities (Maginador et al., 2020). Although the use of %maxHR is a practical method to determine intensity when performing continuous or interval exercise, the physical status of patients with cancer can vary daily due to treatment side effects, which can greatly affect exercise-induced heart rate response. An alternative strategy for monitoring exercise intensity is to assess the RPE. The RPE has a strong association with %maxHR (Scherr et al., 2013). Thus, RPE could be used to monitor training intensity; for instance, values between 12 and 14 on a 6 to 20 scale correspond to 60–80% of VO_2__max_. If one wanted to perform high-intensity exercise sessions (i.e., ≥ 80% of maxHR), targeted RPE values should be ≥ 15 (Singh et al., 2018). Once heart rate and RPE values are within the prescribed ranges during exercise sessions, one should expect increases or maintenance of CRF during the COVID-19 era and after the SARS-CoV-2 vaccine.

Although cardiorespiratory exercises are simple and easy to perform, they require expensive equipment or space and are difficult to perform during home confinement. Alternatively, during COVID-19 physical distancing, body weight, elastic band, and suspension band exercises can also be adapted to maintain or improve CRF while being time-efficient and enjoyable. Individuals can perform large muscle group exercises, either continuously or intermittently, which increase oxygen consumption and, over time, the CRF (Burd et al., 2010; Myers et al., 2015; Tabata, 2019). Several bodyweight (e.g., jumping jacks, butt kicks, squat sidekick), elastic resistance bands (e.g., overhead squat, push-ups), and suspension bands (e.g., squat or a variation to single-leg squat, hamstring curl, low row, and chest press) exercises can be performed even by individuals with low CRF. It is possible to increase the energy demand using higher intensity exercises such as burpees, squat jumps, jump squats, and mountain climbers. For example, performing 8 sets of 20 s of a single exercise per day (e.g., day 1 burpees, day 2 jumping jacks, day 3 mountain climbers) interspaced by 10 s rest periods increased CRF to the same extent as running 30 min at 85% of maxHR after four weeks of training. Likewise, there appear to be no significant differences in CRF gains between bodyweight exercise [e.g., split squat jumps, mountain climber, high knees, burpees (without jump), and jumping jacks] programs and interval running programs (Menz et al., 2019). Importantly, muscle strength and mass also increase with these exercise programs. They only need to perform exercises with body weight, elastic band exercise, and suspension bands to voluntary interruption (i.e., close to concentric muscle failure) to maximize the neuromuscular stimulus. The rest interval between sets and exercises can be short (e.g., 15–30 s) or long (e.g., 1–4 min) (Feito et al., 2018) while alternating the exercise mode, or upper and lower limbs (Myers et al., 2015) and maintaining the %maxHR range during exercise. For example, Myers et al. (2015) trained young women with a traditional training program to increase strength, muscle mass, and CRF, performed with weightlifting machines, cycle ergometer, treadmill, or bodyweight exercise. The researchers reported that a bodyweight exercise program induced superior CRF gains and similar muscle strength gains compared to weight lifting machines, cycle ergometer, and treadmill. Importantly, this type of exercise program can decrease exercise duration and increase motivation, which are likely to increase exercise adherence.

Overall, we recommend that to maintain or increase muscle strength and mass and CRF during COVID-19 physical distancing, exercises performed with bodyweight, elastic bands, or suspension bands should be performed to voluntary interruption, regardless of the target number of repetitions in each set. Additionally, rest intervals between sets and exercises (i.e., long or short) should be manipulated to maintain the intensity of exercise above 50–80% of maxHR and/or 12 of RPE. For an exercise program with these characteristics, the progression of the stimulus must be carried out by increasing the complexity of the exercises, the number of sets, and the weekly frequency.

It is important to emphasize that cancer patients must be frequently monitored regarding their health status, fatigue levels, and well-being to increase the safety and efficiency of the exercise prescription, which must emphasize the patients’ needs and overall health (Hayes et al., 2019). Despite understanding the effectiveness of our recommendations, we hope that after a massive anti-COVID-19 vaccination, cancer patients may exercise in well-equipped facilities and under specialized supervision to increase the efficiency compared to home-based programs (Schmitz et al., 2019).

## Considerations

We provide exercise recommendations for cancer patients, under or beyond treatment, to keep or increase muscle mass and CRF without sophisticated equipment while under home confinement during the COVID-19 era and after the SARS-CoV-2 vaccine. With feasible exercises attainable anywhere, modulating only the work-to-rest ratio and using voluntary interruption, %maxHR, and RPE for monitoring exercise intensity, it is possible to prescribe exercise for a wide range of patients with cancer as well as exercise-induced adaptations. Exercise must be encouraged; however, exercise professionals must be aware of the patient’s health condition even at physical distance to provide a safe and efficient exercise program. Exercise professionals should adjust the exercise prescription throughout home confinement whenever necessary, keeping in mind that minimal exercise stimuli are beneficial to patients in poor physical condition, and prescriptions above the standard recommendation can provide additional benefits to patients in good physical condition. It is important to highlight that the suggestions of the present study were based on data available in the literature; thus, future studies should validate our recommendations. According to the 2020 World Health Organization guidelines (Bull et al., 2020), adults and older adults with chronic conditions such as cancer, should minimize sedentary time as any physical activity is better than none.

## Data Availability Statement

The original contributions presented in the study are included in the article/supplementary material, further inquiries can be directed to the corresponding author.

## Author Contributions

MC conceived the idea, wrote the first draft, worked on all drafts, and formatted the manuscript for submission. SD, FV, GT, GM, LS, CL, and CU helped to develop the main idea and drafting the manuscript. All authors read and approved the final version of the manuscript.

## Conflict of Interest

The authors declare that the research was conducted in the absence of any commercial or financial relationships that could be construed as a potential conflict of interest.

## References

[B1] Acsm (2011). American college of sports medicine position stand. quantity and quality of exercise for developing and maintaining cardiorespiratory, musculoskeletal, and neuromotor fitness in apparently healthy adults: guidance for prescribing exercise. *Med. Sci. Sports Exerc.* 43 1334–1359. 10.1249/mss.0b013e318213fefb 21694556

[B2] Ausoni S. Calamelli S. Sacca S. Azzarello G. (2020). How progressive cancer endangers the heart: an intriguing and underestimated problem. *Cancer Metastasis Rev.* 39 535–552. 10.1007/s10555-020-09869-8 32152913

[B3] Broughman J. R. Williams G. R. Deal A. M. Yu H. Nyrop K. A. Alston S. M. (2015). Prevalence of sarcopenia in older patients with colorectal cancer. *J. Geriatr. Oncol.* 6 442–445. 10.1016/j.jgo.2015.08.005 26365898 PMC7108316

[B4] Bull F. C. Al-Ansari S. S. Biddle S. Borodulin K. Buman M. P. Cardon G. (2020). World Health Organization 2020 guidelines on physical activity and sedentary behaviour. *Br. J. Sports Med.* 54 1451–1462. 10.1136/bjsports-2020-102955 33239350 PMC7719906

[B5] Burd N. A. West D. W. Staples A. W. Atherton P. J. Baker J. M. Moore D. R. (2010). Low-load high volume resistance exercise stimulates muscle protein synthesis more than high-load low volume resistance exercise in young men. *PLoS One* 5:e12033. 10.1371/journal.pone.0012033 20711498 PMC2918506

[B6] Calatayud J. Borreani S. Colado J. C. Martin F. Tella V. Andersen L. L. (2015). Bench press and push-up at comparable levels of muscle activity results in similar strength gains. *J. Strength Cond. Res.* 29 246–253. 10.1519/jsc.0000000000000589 24983847

[B7] Campbell K. L. Winters-Stone K. M. Wiskemann J. Courneya K. S. May A. M. Schwartz A. L. (2019). Exercise guidelines for cancer survivors: consensus statement from international multidisciplinary roundtable. *Med. Sci. Sports Exerc.* 51 2375–2390. 10.1249/mss.0000000000002116 31626055 PMC8576825

[B8] Chang K. V. Chen J. D. Wu W. T. Huang K. C. Hsu C. T. Han D. S. (2018). Association between loss of skeletal muscle mass and mortality and tumor recurrence in hepatocellular carcinoma: a systematic review and meta-analysis. *Liver Cancer* 7 90–103. 10.1159/000484950 29662836 PMC5892377

[B9] Clauss D. Tjaden C. Hackert T. Schneider L. Ulrich C. M. Wiskemann J. (2017). Cardiorespiratory fitness and muscle strength in pancreatic cancer patients. *Support. Care Cancer.* 25 2797–2807. 10.1007/s00520-017-3694-8 28417202

[B10] Damas F. Angleri V. Phillips S. M. Witard O. C. Ugrinowitsch C. Santanielo N. (2019). Myofibrillar protein synthesis and muscle hypertrophy individualized responses to systematically changing resistance training variables in trained young men. *J. Appl. Physiol. (1985)* 127 806–815. 10.1152/japplphysiol.00350.2019 31268828

[B11] de Oliveira P. A. Blasczyk J. C. Souza Junior G. Lagoa K. F. Soares M. de Oliveira R. J. (2017). Effects of elastic resistance exercise on muscle strength and functional performance in healthy adults: a systematic review and meta-analysis. *J. Phys. Act. Health* 14 317–327. 10.1123/jpah.2016-0415 28032811

[B12] Early Breast Cancer Trialists’ Collaborative Group (2005). Effects of chemotherapy and hormonal therapy for early breast cancer on recurrence and 15-year survival: an overview of the randomised trials. *Lancet* 365 1687–1717. 10.1016/s0140-6736(05)66544-0 15894097

[B13] Early Breast Cancer Trialists’ Collaborative Group Darby S. McGale P. Correa C. Taylor C. Arriagada R. (2011). Effect of radiotherapy after breast-conserving surgery on 10-year recurrence and 15-year breast cancer death: meta-analysis of individual patient data for 10,801 women in 17 randomised trials. *Lancet* 378 1707–1716. 10.1016/s0140-6736(11)61629-2 22019144 PMC3254252

[B14] Feito Y. Heinrich K. M. Butcher S. J. Poston W. S. C. (2018). High-intensity functional training (HIFT): definition and research implications for improved fitness. *Sports (Basel)* 6:76. 10.3390/sports6030076 30087252 PMC6162410

[B15] Griffin O. M. Duggan S. N. Ryan R. McDermott R. Geoghegan J. Conlon K. C. (2019). Characterising the impact of body composition change during neoadjuvant chemotherapy for pancreatic cancer. *Pancreatology* 19 850–857. 10.1016/j.pan.2019.07.039 31362865

[B16] Guinan E. M. Doyle S. L. Bennett A. E. O’Neill L. Gannon J. Elliott J. A. (2018). Sarcopenia during neoadjuvant therapy for oesophageal cancer: characterising the impact on muscle strength and physical performance. *Support. Care Cancer* 26 1569–1576. 29197960 10.1007/s00520-017-3993-0

[B17] Haskell W. L. Lee I. M. Pate R. R. Franklin B. A. Powell K. E. Blair S. N. (2007). Physical activity and public health: updated recommendation for adults from the American College Of Sports Medicine and the American Heart Association. *Med. Sci. Sports Exerc.* 39 1423–1434. 10.1249/mss.0b013e3180616b27 17762377

[B18] Hayes S. C. Newton R. U. Spence R. R. Galvao D. A. (2019). The exercise and sports science Australia position statement: exercise medicine in cancer management. *J. Sci. Med. Sport* 22 1175–1199. 10.1016/j.jsams.2019.05.003 31277921

[B19] Kantor B. N. Kantor J. (2020). Non-pharmaceutical interventions for pandemic COVID-19: a cross-sectional investigation of US general public beliefs, attitudes, and actions. *Front. Med. (Lausanne)* 7:384. 10.3389/fmed.2020.00384 32719807 PMC7347901

[B20] Kazemi-Bajestani S. M. Mazurak V. C. Baracos V. (2016). Computed tomography-defined muscle and fat wasting are associated with cancer clinical outcomes. *Semin. Cell Dev. Biol.* 54 2–10. 10.1016/j.semcdb.2015.09.001 26343952

[B21] Khoramipour K. Basereh A. Hekmatikar A. A. Castell L. Ruhee R. T. Suzuki K. (2021). Physical activity and nutrition guidelines to help with the fight against COVID-19. *J. Sports Sci.* 39 101–107. 10.1080/02640414.2020.1807089 32842905

[B22] Kikuchi N. Nakazato K. (2017). Low-load bench press and push-up induce similar muscle hypertrophy and strength gain. *J. Exerc. Sci. Fit.* 15 37–42. 10.1016/j.jesf.2017.06.003 29541130 PMC5812864

[B23] Krogh-Madsen R. Thyfault J. P. Broholm C. Mortensen O. H. Olsen R. H. Mounier R. (2010). A 2-wk reduction of ambulatory activity attenuates peripheral insulin sensitivity. *J. Appl. Physiol. (1985)* 108 1034–1040. 10.1152/japplphysiol.00977.2009 20044474

[B24] Kyle U. G. Morabia A. Schutz Y. Pichard C. (2004). Sedentarism affects body fat mass index and fat-free mass index in adults aged 18 to 98 years. *Nutrition* 20 255–260. 10.1016/j.nut.2003.11.019 14990265

[B25] Lee K. Kang I. Mack W. J. Mortimer J. Sattler F. Salem G. (2019). Feasibility of high intensity interval training in patients with breast cancer undergoing anthracycline chemotherapy: a randomized pilot trial. *BMC Cancer* 19:653. 10.1186/s12885-019-5887-7 31269914 PMC6610838

[B26] Liao C. D. Tsauo J. Y. Chiu Y. S. Ku J. W. Huang S. W. Liou T. H. (2020). Effects of elastic resistance exercise after total knee replacement on muscle mass and physical function in elderly women with osteoarthritis: a randomized controlled trial. *Am. J. Phys. Med. Rehabil.* 99 381–389. 10.1097/phm.0000000000001344 31687984

[B27] Liao C. D. Tsauo J. Y. Huang S. W. Ku J. W. Hsiao D. J. Liou T. H. (2018). Effects of elastic band exercise on lean mass and physical capacity in older women with sarcopenic obesity: a randomized controlled trial. *Sci. Rep.* 8:2317. 29396436 10.1038/s41598-018-20677-7 PMC5797161

[B28] Lopes J. S. S. Machado A. F. Micheletti J. K. de Almeida A. C. Cavina A. P. Pastre C. M. (2019). Effects of training with elastic resistance versus conventional resistance on muscular strength: a systematic review and meta-analysis. *SAGE Open Med.* 7:2050312119831116. 30815258 10.1177/2050312119831116 PMC6383082

[B29] Maginador G. Lixandrao M. E. Bortolozo H. I. Vechin F. C. Sarian L. O. Derchain S. (2020). Aerobic exercise-induced changes in cardiorespiratory fitness in breast cancer patients receiving chemotherapy: a systematic review and meta-analysis. *Cancers (Basel)* 12:2240. 10.3390/cancers12082240 32796499 PMC7463807

[B30] Menz V. Marterer N. Amin S. B. Faulhaber M. Hansen A. B. Lawley J. S. (2019). Functional vs. running low-volume high-intensity interval training: effects on vo2max and muscular endurance. *J. Sports Sci. Med.* 18 497–504. 31427872 PMC6683610

[B31] Mok N. W. Yeung E. W. Cho J. C. Hui S. C. Liu K. C. Pang C. H. (2015). Core muscle activity during suspension exercises. *J. Sci. Med. Sport* 18 189–194. 10.1016/j.jsams.2014.01.002 24556020

[B32] Myers T. R. Schneider M. G. Schmale M. S. Hazell T. J. (2015). Whole-body aerobic resistance training circuit improves aerobic fitness and muscle strength in sedentary young females. *J. Strength Cond. Res.* 29 1592–1600. 10.1519/jsc.0000000000000790 25486302

[B33] Nipp R. D. Fuchs G. El-Jawahri A. Mario J. Troschel F. M. Greer J. A. (2017). Sarcopenia is associated with quality of life and depression in patients with advanced cancer. *Oncologist* 23 97–104. 10.1634/theoncologist.2017-0255 28935775 PMC5759817

[B34] Nobrega S. R. Ugrinowitsch C. Pintanel L. Barcelos C. Libardi C. A. (2018). Effect of resistance training to muscle failure vs. volitional interruption at high- and low-intensities on muscle mass and strength. *J. Strength Cond. Res.* 32 162–169. 10.1519/jsc.0000000000001787 29189407

[B35] Papiol M. Serra-Prat M. Vico J. Salvador N. Garcia M. Camps M. (2016). Poor muscle strength and low physical activity are the most prevalent frailty components in community-dwelling older adults. *J. Aging Phys. Act.* 24 363–368. 10.1123/japa.2015-0114 26540738

[B36] Psutka S. P. Carrasco A. Schmit G. D. Moynagh M. R. Boorjian S. A. Frank I. (2014). Sarcopenia in patients with bladder cancer undergoing radical cystectomy: impact on cancer-specific and all-cause mortality. *Cancer* 120 2910–2918. 10.1002/cncr.28798 24840856

[B37] Ralston G. W. Kilgore L. Wyatt F. B. Baker J. S. (2017). The effect of weekly set volume on strength gain: a meta-analysis. *Sports Med.* 47 2585–2601. 10.1007/s40279-017-0762-7 28755103 PMC5684266

[B38] Reisinger K. W. Bosmans J. W. Uittenbogaart M. Alsoumali A. Poeze M. Sosef M. N. (2015). Loss of skeletal muscle mass during neoadjuvant chemoradiotherapy predicts postoperative mortality in esophageal cancer surgery. *Ann. Surg. Oncol.* 22 4445–4452. 10.1245/s10434-015-4558-4 25893413 PMC4644199

[B39] Santanielo N. Scarpelli M. Alvarez I. E. Otoboni G. B. Pintanel L. Libardi C. A. (2020). Effect of resistance training to muscle failure vs non-failure on strength, hypertrophy and muscle architecture in trained individuals. *Biol. Sport* 37 333–341. 10.5114/biolsport.2020.96317 33343066 PMC7725035

[B40] Scherr J. Wolfarth B. Christle J. W. Pressler A. Wagenpfeil S. Halle M. (2013). Associations between Borg’s rating of perceived exertion and physiological measures of exercise intensity. *Eur. J. Appl. Physiol.* 113 147–155. 10.1007/s00421-012-2421-x 22615009

[B41] Schmitz K. H. Troxel A. B. Dean L. T. DeMichele A. Brown J. C. Sturgeon K. (2019). Effect of home-based exercise and weight loss programs on breast cancer-related lymphedema outcomes among overweight breast cancer survivors: the wiser survivor randomized clinical trial. *JAMA Oncol.* 5 1605–1613. 10.1001/jamaoncol.2019.2109 31415063 PMC6696732

[B42] Schoenfeld B. J. Ogborn D. Krieger J. W. (2016). Dose-response relationship between weekly resistance training volume and increases in muscle mass: a systematic review and meta-analysis. *J. Sports Sci.* 35 1073–1082. 10.1080/02640414.2016.1210197 27433992

[B43] Scott J. M. Zabor E. C. Schwitzer E. Nilsen T. S. Koelwyn G. J. Adams S. C. (2018). Efficacy of exercise therapy on cardiorespiratory fitness in patients with cancer: a systematic review and meta-analysis. *J. Clin. Oncol.* 36 2297–2305. 10.1200/jco.2017.77.5809 29894274 PMC6804903

[B44] Singh B. Spence R. R. Steele M. L. Sandler C. X. Peake J. M. Hayes S. C. A. (2018). Systematic review and meta-analysis of the safety, feasibility, and effect of exercise in women with stage II+ breast cancer. *Arch. Phys. Med. Rehabil.* 99 2621–2636. 10.1016/j.apmr.2018.03.026 29730319

[B45] Soligon S. D. da Silva D. G. Bergamasco J. G. A. Anglieri V. Júnior R. A. M. Dias N. F. (2020). Suspension training vs. traditional resistance training: efects on muscle mass, strength and functional performance in older adults. *Eur. J. Appl. Physiol.* 120 2223–2232. 10.1007/s00421-020-04446-x 32700098

[B46] Stanton R. To Q. G. Khalesi S. Williams S. L. Alley S. J. Thwaite T. L. (2020). Depression, anxiety and stress during COVID-19: associations with changes in physical activity, sleep, tobacco and alcohol use in australian adults. *Int. J. Environ. Res. Public Health* 17:4065. 10.3390/ijerph17114065 32517294 PMC7312903

[B47] Tabata I. (2019). Tabata training: one of the most energetically effective high-intensity intermittent training methods. *J. Physiol. Sci.* 69 559–572. 10.1007/s12576-019-00676-7 31004287 PMC10717222

[B48] van der Kolk N. M. de Vries N. M. Kessels R. P. C. Joosten H. Zwinderman A. H. Post B. (2019). Effectiveness of home-based and remotely supervised aerobic exercise in Parkinson’s disease: a double-blind, randomised controlled trial. *Lancet Neurol.* 18 998–1008. 10.1016/s1474-4422(19)30285-6 31521532

[B49] van der Leeden M. Huijsmans R. J. Geleijn E. de Rooij M. Konings I. R. Buffart L. M. (2018). Tailoring exercise interventions to comorbidities and treatment-induced adverse effects in patients with early stage breast cancer undergoing chemotherapy: a framework to support clinical decisions. *Disabil. Rehabil.* 40 486–496. 10.1080/09638288.2016.1260647 28054496

[B50] Villasenor A. Ballard-Barbash R. Baumgartner K. Baumgartner R. Bernstein L. McTiernan A. (2012). Prevalence and prognostic effect of sarcopenia in breast cancer survivors: the heal study. *J. Cancer Surviv.* 6 398–406. 10.1007/s11764-012-0234-x 23054848 PMC3747827

[B51] Wallen M. P. Hennessy D. Brown S. Rawstorn C. J. Hall A. (2020). High-intensity interval training improves cardiorespiratory fitness in cancer patients and survivors: a meta-analysis. *Eur. J. Cancer Care* 29:e13267. 10.1111/ecc.13267 32469144

[B52] Wang Y. Chen S. Zhang J. Zhang Y. Ernstsen L. Lavie C. J. (2018). Nonexercise estimated cardiorespiratory fitness and all-cancer mortality: the nhanes iii study. *Mayo Clin. Proc.* 93 848–856. 10.1016/j.mayocp.2018.01.004 29602418

